# Use of mindfulness in the treatment of tinnitus: consensus based on the opinion of Brazilian experts

**DOI:** 10.1590/2317-1782/e20250182en

**Published:** 2026-07-06

**Authors:** Mônica Claudino Medeiros Honorato, Mariana Lopes Martins, Lidiane Maria de Brito Macedo Ferreira, Erika Baroni Mantello, Marine Raquel Diniz da Rosa

**Affiliations:** 1 Universidade Federal da Paraíba – UFPB - João Pessoa (PB), Brasil.; 2 Departamento de Fonoaudiologia, Universidade Federal da Paraíba – UFPB - João Pessoa (PB), Brasil.; 3 Universidade Federal do Rio Grande do Norte – UFRN - Natal (RN), Brasil.; 4 Curso de Fonoaudiologia, Departamento de Ciências da Saúde, Faculdade de Medicina de Ribeirão Preto, Universidade de São Paulo (USP) – Ribeirão Preto-São Paulo (SP), Brasil.

**Keywords:** Tinnitus, Mindfulness, Delphi Technique, Consensus, Complementary Therapies

## Abstract

**Purpose:**

To develop a consensus among otolaryngologists, speech-language-hearing pathologists, and physical therapists regarding the criteria for recommending and using mindfulness in the treatment of tinnitus.

**Methods:**

Observational, descriptive, quantitative, cross-sectional study conducted online. The convenience sample initially consisted of 23 specialists. The Delphi method was applied in two stages, providing feedback with anonymous responses. The first stage consisted of objective and subjective questions. The second stage involved 10 participants, who evaluated 34 statements derived from the initial stage. The specialists analyzed each item and demonstrated their agreement on a 5-point Likert scale. The content validity coefficient (CVC) was used to investigate the degree of interrater agreement and select the final items.

**Results:**

There was consensus on 25 of the 34 items (CVC ≥ 0.70). The specialists consider mindfulness to be a therapeutic resource for tinnitus, but not as monotherapy. There was consensus on recommending application once a week, for at least 30 minutes, with daily home practice. Follow-up therapy after 8 weeks can be done using scales such as the Tinnitus Handicap Inventory and the Visual Analogue Scale.

**Conclusion:**

There is consensus on the combined (not alone) use of mindfulness in the treatment of tinnitus with a weekly protocol (8 weeks), lasting 30 minutes per session, monitored using the THI and VAS. These recommendations should be explored in clinical trials, investigating the short- and long-term effects to provide scientific evidence and standardize clinical protocols.

## INTRODUCTION

Tinnitus is a symptom defined as the conscious perception of sound in the absence of an external generating source^(1)^. Its prevalence varies according to populations and studies. A systematic review and meta-analysis indicated a global prevalence of tinnitus in adults of approximately 14.4%, with an increase associated with age^(2)^.

Tinnitus may be associated with approximately 300 clinical conditions^(3)^, including otologic diseases, head and neck trauma, cardiovascular disorders, metabolic, neurological, and psychiatric diseases, dental factors, medication side effects, and drug abuse.

The impact of tinnitus is heterogeneous^(4)^; most people are not bothered by it, and approximately a quarter of patients seek medical attention^(1)^. The condition may be classified as a tinnitus disorder in the presence of emotional disturbance, cognitive impairment, and/or autonomic arousal, resulting in behavioral changes and functional disability^(5)^.

An individualized assessment can significantly influence adherence to and success in tinnitus treatment^(6)^, especially when conducted in a multidisciplinary context (such as otolaryngology, speech-language-hearing therapy, physiotherapy, and psychology) with complementary perspectives.

Identifying specific patient factors allows for targeted interventions that improve quality of life and, consequently, adherence to treatment. The clinical heterogeneity of tinnitus requires the stratification of subgroups of patients who respond more predictably to specific treatments^(7)^.

In some cases, tinnitus counseling is sufficient to restore patients' quality of life. However, if there is no significant improvement within 2 months, additional treatments should be proposed, either through a single therapy or by combining more than one^(8)^.

The practice of mindfulness has been investigated as a therapeutic option for the treatment of tinnitus, especially for reducing stress and distress associated with the condition. It is defined as the effort to be intentionally attentive, without judgment, on a moment-to-moment basis^(4)^.

It is believed that tinnitus distress can be generated by negative thoughts and attempts at avoidance. Mindfulness works on habituation and acceptance and has proven to be an effective non-pharmacological intervention^(9)^.

Most of the time, the main challenge in tinnitus treatment is to alleviate the distress associated with the symptom. Mindfulness-based approaches can offer a new way of dealing with it, being quite distinct from conventional audiological treatments^(10)^.

Mindfulness was introduced into modern Western medicine by Jon Kabat-Zinn to treat chronic pain, but it has now proven effective in treating stress, anxiety, and depression^(4)^. Different approaches include Mindfulness-Based Stress Reduction (MBSR), Mindfulness-Based Cognitive Therapy (MBCT), and Mindfulness-Based Health Promotion (MBHP).

Applying this technique to tinnitus has brought benefits, such as reducing the severity of the symptom, psychological suffering, and disability^(11)^, and decreasing the distress related to tinnitus^(9)^.

The practice of mindfulness can help mitigate the negative effects of ironic mind control, in which the attempt to suppress emotional sounds can, paradoxically, increase the salience and persistence of tinnitus. Thus, it helps patients develop an open and stable awareness, enabling them to "allow" the presence of tinnitus without fighting against it, which can reduce the intrusiveness and emotional impact of tinnitus^(12)^.

This therapy offers a non-invasive, accessible, and potentially effective approach to managing chronic tinnitus, with benefits that include reducing suffering and improving the overall well-being of patients^(4,13)^.

However, the protocols used are highly variable, so it is necessary to establish a consensus that defines which patient groups are best candidates for mindfulness in the treatment of tinnitus, the average number of sessions needed for symptom remission, the recommended frequency, and how to follow up these patients.

One way to create consensus is through the Delphi method^(14,15)^. This procedure structures communication between a group of experts on complex issues, through interactions carried out by questionnaires. These are accompanied by feedback via email, maintaining the anonymity of the participants' responses, in the search for a common result, through statistical calculations^(14)^.

This method gathers a set of opinions from geographically separated experts, which can favor consistent results on complex topics^(16)^. The Delphi method is considered important in scientific health research, as consensus can be used to direct scientific research^(14)^.

Thus, this research aimed to develop a consensus with otolaryngology, speech-language-hearing, and physiotherapy experts regarding the criteria for recommending and using mindfulness applied to the treatment of tinnitus. It also aimed to verify their knowledge regarding the use of mindfulness in the treatment of tinnitus, identify which patient groups would be the best candidates for mindfulness in the treatment of tinnitus, the average number of sessions needed for symptom remission and their frequency, and how to follow up these patients.

## METHODS

This is an observational, descriptive, quantitative, cross-sectional study. It was based on the recommendations of Strengthening the Reporting of Observational Studies in Epidemiology (STROBE) and approved by the Research Ethics Committee of the Health Sciences Center (CCS) under approval number 6,666,318.

The research was conducted remotely and online, through a web survey hosted on Google Forms, generating greater convenience and speed and reducing costs. Data were collected between June and December 2024.

The sample was by convenience; the first stage initially included 23 Brazilian specialists (otolaryngologists, speech-language-hearing pathologists, and physiotherapists) with recognized knowledge about the subject under study. Professionals who met at least one of the following criteria were selected for the sample: (a) publication of articles in the area of ​​tinnitus in the last 10 years, (b) participation in a research project in this area in the last 5 years, (c) clinical practice in the area, (d) specialization related to tinnitus, and/or (e) knowledge or use of mindfulness practice for tinnitus treatment.

In the second stage, the questionnaire was sent to 11 of the 23 specialists (10 of them responded), as the eligibility criterion was those who actually applied mindfulness in their daily practices. Among those eligible, seven based their practice on a specific mindfulness approach, while three did not follow any specific protocol. The others only recommend that patients do so and refer them to specialized professionals, which could pose a confounding bias in our study.

**Figure 1:**
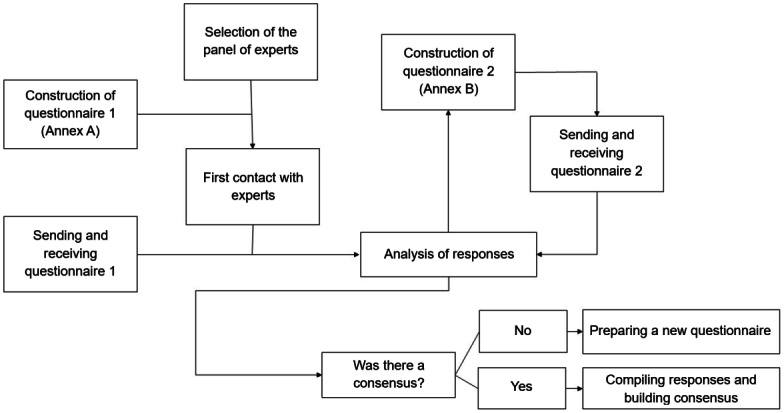
Research stages

The research used the Delphi method to reach consensus among specialists on the use of mindfulness in patients with tinnitus. The procedures had several steps, described in Figure 1.

Caption: Mcvc_i_ = Mean of the content validity coefficients of the questionnaire items; Mpe_i_ = mean of the errors of the questionnaire items

The development of the items for the first questionnaire was preceded by a literature review. The data collection instrument applied in the first stage included subjective and objective questions that covered data on the participants' professional profile and 14 questions about the use of mindfulness in the treatment of tinnitus.

Before answering the questions, participants were contacted and given access to the explanatory text about the research. Then, they proceeded to read the informed consent form and, if they agreed, they continued with the answers. After receiving the answers to questionnaire 1, they underwent qualitative and quantitative analysis and review.

The second stage included the construction of questionnaire 2 and feedback via email to the participants on the questions from the first stage. The objective was to understand how professionals use mindfulness for the treatment of tinnitus in their clinical practices. All 10 participants in the second stage had training in mindfulness, but seven based their practice on some specific mindfulness approach, and three did not follow any specific protocol.

All 34 questions in questionnaire 2 were objective, consisting of statements that emerged from the responses of the first stage. These statements were entered into a form in Google Forms, and the experts were asked to rate them using the 5-point Likert scale, which offers response options ranging from "strongly disagree" (1 point) to "strongly agree" (5 points) (Marques; Freitas, 2018).

After answering all the questions, the expert clicked "Submit," and the data collection procedure was completed. The time to complete the form was estimated at about 10 minutes. The instrument was available for responses between November 5, 2024, and December 6, 2024.

The responses from the 10 experts to the 34 questions were organized in a Microsoft Excel spreadsheet for a consensus analysis. A content validity coefficient (CVC) analysis was performed to verify the interrater agreement in evaluating each item of the questionnaire, as proposed by Hernández-Nieto (Hernández-Nieto, 2002). With the CVC calculation, the experts’ average scores on the Likert scale (1 to 5 points per item) were obtained.

**Figure 2:**
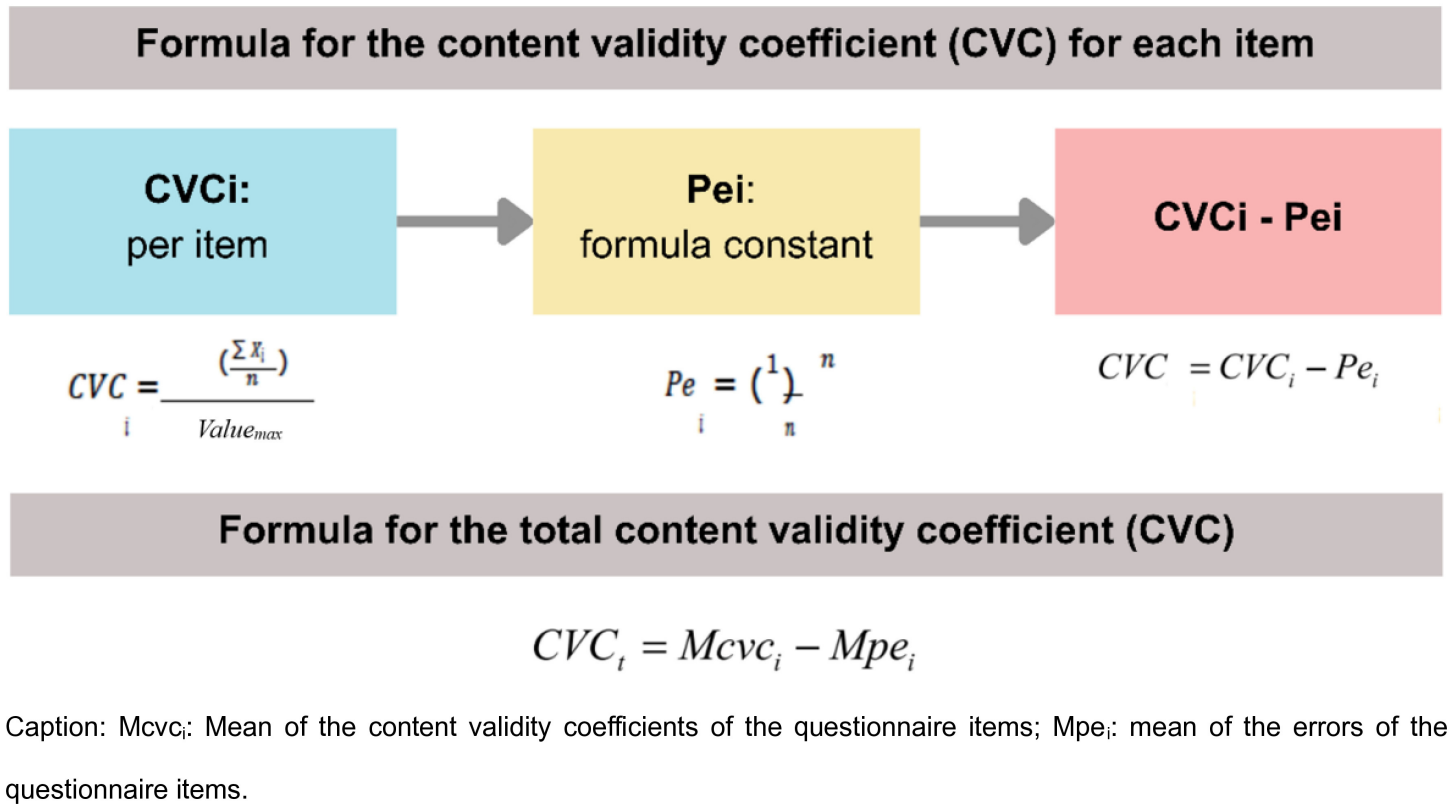
Formula used to calculate the content validity coefficient (CVC)

Source: Honorato (2025)

The average of the attributions per item, the total, and the error were calculated for analysis. The final calculation used the CVC of each item, subtracted by the constant of the formula or error calculation (Figure 2). The final CVC values ​​were classified as (a) excellent (CVI ≥ 0.78), (b) good (0.60 ≥ CVI ≤ 0.77), and (c) poor (CVI ≥ 0.59) (I. American Psychological Association. II. National Council on Measurement in Education. III. Joint Committee on Standards for Educational and Psychological Testing [U.S.], 2014).

There are several definitions for consensus (percentage of agreement, measure of central tendency, dispersion of responses, and so forth), with different levels for each one. This research used the percentage of agreement above 70%, which is widely considered rigorous^(14)^.

## RESULTS

Twenty-three experts participated in this research (questionnaire 1), whose information can be found in Table 1.

**Table 1:** Professional characteristics of the experts

**Variable**	**N**	**%**
**Occupation**		
Otolaryngologist	10	43.4
Speech-language-hearing pathologist	10	43.4
Physiotherapist	3	13
**Sex**		
Females	20	86.9
Males	3	13
**Age range**		
31 to 40 years	10	43.4
41 to 50 years	7	30.4
51 to 60 years	6	26
**Education level**		
Specialization	7	30.4
Master’s degree	4	17.3
Doctoral degree	9	39.1
Postdoctoral degree	3	13
	23	100

Source: Honorato (2025)

In the first stage, all participants were aware of the application of mindfulness in the treatment of tinnitus, but only 34.78% had training in this specific area.

When asked if the interviewee had publications or experience in the treatment of tinnitus, 86.9% answered yes, while three people (13.1%) had knowledge of the practice of the mindfulness technique.

The majority (82.6%) of those interviewed recommend mindfulness in their clinical practice for the treatment of tinnitus, but only 11 (47.8%) actually apply the technique in their daily practice.

Although all experts affirm that this therapeutic resource is indicated for the treatment of tinnitus, 56.5% do not consider that it can be applied to all types. When asked about the three main causes of tinnitus for which specialists most often use or recommend mindfulness, the most frequent answers were tinnitus associated with emotional causes or depressive or anxious states, somatosensory tinnitus, metabolic tinnitus, tinnitus associated with hearing loss, and so on.

Moreover, 82.6% of respondents do not use mindfulness as the sole treatment (monotherapy) for this indication. The most cited complementary therapies were counseling, including dietary guidance, lifestyle changes, and comorbidity management, auditory rehabilitation, sound therapy, physiotherapy treatment, and medication use, among others.

Of the 11 specialists who apply mindfulness in their clinical practice, the following results were obtained (questionnaire 2) regarding the protocol they use, the number of sessions per week, the duration of the sessions, the number of exercises they do in each session, the minimum number of sessions to achieve remission, and how they follow up with patients (Table 2).

**Table 2:** Responses from experts who apply mindfulness (questionnaire 1)

	**Protocol**	**Weekly sessions**	**Session duration (minutes)**	**Exercises per session**	**Minimum number of sessions for remission**	**Follow-up**
1	MBCT	1	50	2	8	THI.
2	MBSR	1	60	2	8	Guidance and recommendations on how to deal with challenges using mindfulness tools
3	MBSR	7	10 to 15	1	I do not know	I do not have a follow-up protocol for mindfulness.
4	MBHP	2	60	3	8	In addition to performing some mindfulness exercises in the office, the patient continues with indirect mindfulness guidelines to practice at home, where I try to change the stimulus every week.
5	None	7	60	It depends; each session is unique	I do not know	I do not specifically know
6	None	1	40	1	8	Visit once a week with home care instructions, for 8 weeks
7	MBCT	1	45	2 to 5	6 to 8	Patients keep the audio recordings and all the written instructions. I suggest they maintain the daily changes and at least one daily meditation
8	None	1	30	2	4	THI and VAS
9	MBCT	1	60 to 90	3 to 4	I do not know	Weekly
10	MBCT	1	40	1 to 2	8	Weekly follow-up with home exercises and guidance for use after the last session
11	MBCT	1	50	2	8	Strategies for using mindfulness in daily life

In the second stage of the Delphi method, responses were obtained from 10 of the 11 specialists who used mindfulness in practice. Considering that the objective of this research was to develop a consensus regarding the use of mindfulness in the treatment of tinnitus, only items with a CVC above 0.70 were considered a consensus^(14)^. The results show a consensus in 25 of the 34 items (CVC ≥ 0.70), which can be verified in Table 3.

**Table 3:** Content validity coefficient (CVC) values ​​per questionnaire item

ITEM	Item CVC
I believe that mindfulness is a suitable therapeutic resource for tinnitus.	0.979
I believe that mindfulness can be applied to all types of tinnitus.	0.739
I do NOT believe that mindfulness can be applied to all types of tinnitus.	0.519
I believe that mindfulness can be applied to tinnitus caused by emotional or psychogenic factors or related to mood disorders.	0.979
I believe that mindfulness can be applied to somatosensory tinnitus.	0.859
I believe that mindfulness can be applied to tinnitus caused by temporomandibular joint (TMJ) disorder.	0.859
I believe that mindfulness can be applied to tinnitus due to hearing loss.	0.899
I believe that mindfulness can be applied to tinnitus that disrupts or disturbs sleep and daily activities.	0.979
I believe that mindfulness can be applied to tinnitus caused by tensor tympani syndrome.	0.819
I believe that mindfulness can be applied to tinnitus that lasts longer than 6 months, regardless of the cause.	0.859
I believe that mindfulness can be applied to pulsatile tinnitus.	0.739
I believe that mindfulness CANNOT be used as the sole therapy for tinnitus (monotherapy).	0.819
I believe that mindfulness can be used as the sole therapy for tinnitus (monotherapy).	0.459
Treatment can combine mindfulness with counseling or psychoeducation about tinnitus.	0.979
Treatment can combine mindfulness with cognitive behavioral therapy.	0.979
Treatment can combine mindfulness with psychotherapy.	0.979
Treatment can combine mindfulness with photobiomodulation.	0.939
Treatment can combine mindfulness with tDCS (transcranial direct current stimulation).	0.939
Treatment can combine mindfulness with TRT (tinnitus retraining therapy)	0.939
Treatment can combine mindfulness with auditory training.	0.939
Treatment can combine mindfulness with physiotherapy techniques (manual therapy, vagal stimulation).	0.959
Treatment can combine mindfulness with neuromodulation	0.959
Treatment can combine mindfulness with medication therapy.	0.959
I apply mindfulness once a week with the professional and provide guidance for daily home practice.	0.899
I apply mindfulness twice a week with the professional and provide guidance for daily home practice.	0.359
Mindfulness sessions last about 1 hour.	0.579
Mindfulness sessions last about 30 minutes.	0.739
I perform one exercise per session.	0.739
I perform two exercises per session.	0.679
I perform three or more exercises per session.	0.519
Remission requires at least 4 sessions.	0.339
Remission requires at least 6 sessions.	0.479
Remission requires at least 8 sessions.	0.659
Therapy follow-up after 8 weeks can use scales such as the THI (Tinnitus Handicap Inventory) and VAS (Visual Analogue Scale).	0.979

Source: Honorato (2025)

## DISCUSSION

Consensus methods assist clinical decision-making by defining criteria in scenarios with limited external evidence or uncertainty about effectiveness. Tinnitus treatment is a clinical challenge because, in addition to the multiple possibilities of etiologies and interventions, it must consider the impact on the patient's quality of life^(17)^.

Mindfulness has proven to be an effective treatment for tinnitus, but there is variability in protocols regarding the number of sessions and follow-up strategies for these patients^(8,11,18)^. This work aimed to develop a consensus to refine recommendations regarding the use of this strategy in the treatment of tinnitus.

### Items agreed upon by experts

#### 1) Best candidates for mindfulness in tinnitus treatment

There was consensus among experts that mindfulness is a therapeutic resource indicated for tinnitus (CVC 0.979). A systematic review evaluated the effectiveness of mindfulness-based interventions in relieving distress caused by tinnitus and found a significant reduction in tinnitus-related distress^(4)^; also, MBCT was associated with greater acceptance of the symptom^(10)^.

It was also agreed that mindfulness can be applied to all types of tinnitus (CVC 0.739), without distinction of sound characteristic, discomfort, or duration. In descending order of agreement levels, we had tinnitus due to emotional or psychogenic causes or related to mood disorders and tinnitus that causes impact or disturbances of sleep and daily activities (CVC 0.979), tinnitus due to hearing loss (CVC 0.899), somatosensory tinnitus due to temporomandibular joint disorder, tinnitus that lasts longer than 6 months, regardless of the cause (CVC 0.859), tinnitus due to tensor tympani syndrome (CVC 0.819), and pulsatile tinnitus (CVC 0.739).

Although studies dealing with the use of mindfulness in the treatment of tinnitus explore little of its specific cause, they indicate that this therapy can be applied to different types of tinnitus, as long as the focus is on relieving psychological suffering and improving the quality of life of patients. However, therapeutic approaches directed at suspected etiologies are closer to successful treatment^(19)^.

On the other hand, the effectiveness of these interventions does not seem to be limited by specific characteristics of tinnitus, such as its origin or type, but rather by the patient's ability to engage in mindfulness practice and develop a new relationship with tinnitus^(11,12)^.

#### 2) Associated therapies

There was agreement that mindfulness cannot be used as the sole therapy for tinnitus (CVC 0.819). Combining therapies for tinnitus treatment can offer superior benefits compared to isolated therapies, especially when customized for patient characteristics. This was demonstrated by a meta-analysis^(20)^, which indicated that combined therapy, such as sound stimulation associated with educational consultation or drug therapy, had significantly better results in reducing tinnitus than individual treatments.

Furthermore, combined therapies seem more suitable in the treatment of tinnitus, since the evidence is often insufficient to support a single treatment method. Also, different approaches add up and complement the mechanisms by which they improve tinnitus^(21)^.

Of the therapies that can be associated with mindfulness, tinnitus counseling or psychoeducation, cognitive behavioral therapy, and psychotherapy obtained the highest agreement index (CVC 0.979). They were followed by physiotherapy maneuvers (manual therapy, vagal stimulation), neuromodulation, and drug therapy (CVC 0.959); photobiomodulation, transcranial direct current stimulation (tDCS), tinnitus retraining therapy (TRT), and auditory training (CVC 0.939).

Tinnitus psychoeducation provides patients with information about the nature of tinnitus, its possible causes, and the best ways to deal with it. In some cases, this therapy is sufficient to restore a normal quality of life in people with tinnitus, making other interventions unnecessary^(8)^. The German consensus on chronic tinnitus suggests that patient psychoeducation should be the starting point for any therapeutic intervention based on an exhaustive diagnostic assessment^(22)^.

Therapeutic approaches that include counseling, such as cognitive behavioral therapy (CBT), acceptance and commitment therapy (ACT), mindfulness, and tinnitus retraining therapy (TRT), have demonstrated greater effectiveness in managing tinnitus and are therefore recommended as a priority^(22,23)^.

Moreover, sound therapies, such as the use of maskers or hearing aids, are frequently recommended. However, there are variations in guidelines on the use of medications and alternative therapies, reflecting the lack of conclusive evidence in some areas^(22,23)^.

Manual therapies and physiotherapy are promising strategies in cases where tinnitus is associated with temporomandibular joint disorder or cervical problems, being indicated within a multidisciplinary and personalized approach^(24)^.

#### 3) Frequency, duration, and quantity of exercises

Regarding the frequency of application of this technique, consensus was reached on the indication of once a week with daily home practice. All protocols the specialists cited in the first stage use weekly sessions with the professional, as well as guidance to perform daily home exercises, totaling 8 weeks^(25)^.

In randomized clinical trials that investigated the effectiveness of MBCT for the treatment of tinnitus, there were variations in the duration of treatment. In the study by McKenna et al. , MBCT was administered in 8 weekly sessions lasting 120 minutes each^(10)^. Gans et al. also maintained the structure of 8 sessions, but with durations of 150 minutes each^(26)^.

On the other hand, the studies by Philippot et al. and Arif et al. used different formats, with six and five weekly mindfulness sessions, respectively^(8,18)^. These approaches in more condensed periods also showed benefits in reducing the psychological difficulties associated with tinnitus.

Regarding the duration of the sessions, the consensus was approximately 30 minutes. In the MBSR and MBCT protocols, the suggested session duration is 2 to 2 and a half hours. In MBHP, the duration is 90 minutes and includes daily 45-minute meditation practices as a fundamental part of the process^(25)^.

Although the consensus was approximately 30-minute sessions, most researchers in the first stage of the study responded that their sessions usually last an average of 49 minutes (as seen in Table 2). Thus, it is understood that the therapy should have a minimum duration of 30 minutes.

Regarding the number of exercises per session, item 1, exercises per session, obtained an index of 0.739. The RCTs by Arif et al. and Philippot et al. provided the best specification of exercises performed per session among the studies that use mindfulness in the treatment of tinnitus.

The first^(18)^ describes that classes began with a 40-minute meditation practice, followed by a review of the previous week's homework, presentation, and discussion of the session's topic, and ended with a shorter experimental exercise and a review of the homework for the following week.

The second^(8)^ detailed in a table everything that was done in each session, which began with a 30-minute exercise and continued with a review of the previous week's homework, before presenting and discussing the specific topic of the session. Finally, all sessions ended with experimental exercises.

#### 4) Patient follow-up

It was also agreed that therapy follow-up can use scales such as the Tinnitus Handicap Inventory (THI) and Visual Analogue Scale (VAS).

Tinnitus assessment methods are essential tools for characterizing it and documenting the effectiveness of treatments. Currently, the most common are THI and VAS. The THI has the advantage of being a more comprehensive assessment, while the VAS is simpler and easier to understand^(27)^.

THI is a questionnaire composed of 25 items that assess the impact of tinnitus in three dimensions: functional, emotional, and catastrophic. It was developed to measure the severity of tinnitus and the degree of discomfort it causes, providing a total score that reflects the overall severity of the tinnitus. Studies indicate that the THI is a reliable scale^(28)^.

VAS is a simple and quick tool that measures the subjective suffering caused by tinnitus. It assesses aspects such as sound intensity, tinnitus awareness, discomfort, and impact on the patient's life and is effective in capturing rapid changes in tinnitus perception over time^(29)^. It is also considered useful for an initial assessment of tinnitus severity and has shown good correlation with other measures of tinnitus severity, such as the THI^(30)^.

Gans et al. used the THI as a primary outcome measure in their study and considered it psychometrically robust and easy to administer and interpret. They also included VAS and other questionnaires for secondary outcome measures.

Mckenna et al. and Arif et al. used VAS among the questionnaires that measure the perceived severity of tinnitus and post-treatment improvements in the mindfulness-treated groups and in the control groups. Philippot et al. used a questionnaire that was not published and not validated at the time of its publication.

Roland et al. conducted an uncontrolled clinical trial involving 13 patients with bothersome chronic tinnitus who underwent MBSR. The primary outcome measure was the difference in patient-reported tinnitus symptoms using the THI and the Tinnitus Functional Index (TFI) between pre-intervention, post-MBSR, and 4 weeks post-MBSR assessments^(13)^.

This study did not assess duration among specialists. Philippot et al. and Arif et al. followed patients for 3 months. McKenna et al. did so after 1 and 6 months, demonstrating that the effects of mindfulness persisted for long periods.

A study evaluated 80 patients with complete tinnitus remission, following up patients every 6 months until completing 18 months. During this period, 92.1% of participants remained asymptomatic, while only 7.9% reported symptom recurrence^(21)^. These findings encourage a more optimistic approach to managing patients suffering from tinnitus, suggesting that personalizing therapeutic strategies can lead to success in many cases.

The most recent guidelines published for tinnitus management on various continents do not address recommendations on how to follow up these patients^(17,22,23)^. Thus, according to the consensus, we believe it is important to monitor patients using at least the THI and VAS.

### Items without a consensus among experts

There is no consensus among experts on the possibility of tinnitus remission or the minimum number of sessions required. It is important to highlight that the definition of remission in the literature is controversial. Gouveia considered that remission occurs when a patient who had tinnitus daily for more than 3 months ceased to perceive it for at least 6 months^(31)^. However, such a definition may not be applicable to all therapeutic contexts, especially those focused on symptom management approaches, as is the case with mindfulness.

Moreover, the therapeutic response varies significantly among patients, and some patients achieve habituation to the symptom instead of its complete elimination. Another relevant factor is that most experts do not apply mindfulness as the sole therapy, so successful cases may be attributed to other techniques or a combination thereof.

The study has limitations in that the responses were limited to the opinion of some experts; moreover, they were asked closed questions, limiting the discussion on the subject. This decision was made to expand data collection without limiting geographical barriers and to facilitate participation.

Another limitation is the need for a third stage with the topics that did not reach agreement, to try to broaden the level of consensus through feedback and evaluations from each individual. This interaction was not possible due to sample fatigue. The items that say the opposite of the topics already discussed did not obtain consensus, as reinforced by the literature and the consensus on what should be used.

## CONCLUSION

Mindfulness is a therapeutic resource indicated for tinnitus, but not alone. Regarding the frequency of application of this technique, consensus was reached on the recommendation of once a week with a therapist, lasting at least 30 minutes, in addition to daily home practice, with one exercise per session. Moreover, the consensus was that the therapy follow-up after 8 weeks can be evaluated through questionnaires and/or scales, such as the THI and VAS. The only topic on which there was no consensus was the minimum number of sessions to allow for possible remission of tinnitus.
