# Supplementary material for: Use of mindfulness in the treatment of tinnitus: consensus based on the opinion of Brazilian experts
Source: Codas. 2026 Jul 6;38(4):e20250182. doi: 10.1590/2317-1782/e20250182en (PMC13343791; doi:10.1590/2317-1782/e20250182en)
Supplement: Translation

# Translation: Uso da mindfulness no tratamento do zumbido: consenso baseado na opinião de especialistas brasileiros

**DOI:** 10.1590/2317-1782/e20250182pt

**Authors:** Mônica Claudino Medeiros Honorato, Mariana Lopes Martins, Lidiane Maria de Brito Macedo Ferreira, Erika Baroni Mantello, Marine Raquel Diniz da Rosa

**Affiliations:** 1 Universidade Federal da Paraíba – UFPB - João Pessoa (PB), Brasil.; 2 Departamento de Fonoaudiologia, Universidade Federal da Paraíba – UFPB - João Pessoa (PB), Brasil.; 3 Universidade Federal do Rio Grande do Norte – UFRN - Natal (RN), Brasil.; 4 Curso de Fonoaudiologia, Departamento de Ciências da Saúde, Faculdade de Medicina de Ribeirão Preto, Universidade de São Paulo (USP) – Ribeirão Preto-São Paulo (SP), Brasil.

**Keywords:** Zumbido, Atenção Plena, Técnica Delphi, Consenso, Terapias Complementares

## Abstract

**Objetivo:**

Desenvolver um consenso com experts otorrinolaringologistas, fonoaudiólogos e fisioterapeutas acerca dos critérios de recomendação e uso da mindfulness aplicada no tratamento do zumbido.

**Método:**

Estudo observacional, descritivo, quantitativo e transversal, conduzido de forma online. A amostra por conveniência contou, inicialmente, com 23 especialistas. Aplicou-se o método Delphi em duas etapas, com fornecimento de feedback e anonimato das respostas. A primeira etapa consistiu em perguntas objetivas e subjetivas. A segunda etapa contou com 10 participantes, que avaliaram 34 afirmativas derivadas da etapa inicial. Os especialistas analisaram cada item e demonstraram sua concordância em uma escala likert de cinco pontos. Utilizou-se o coeficiente de validade de conteúdo (CVC) para investigar o grau de concordância entre os juízes e selecionar os itens finais.

**Resultados:**

Houve consenso em 25 dos 34 itens (CVC ≥ 0,70). Os especialistas consideram que a mindfulness é um recurso terapêutico para o zumbido, porém não como monoterapia. Foi consenso a indicação da aplicação uma vez por semana, pelo menos, 30 minutos, com prática diária domiciliar. O seguimento da terapia após 8 semanas pode ser feito por meio de escalas como Tinnitus Handicap Inventory e a Escala Visual Analógica.

**Conclusão:**

Há consenso sobre o uso da mindfulness no tratamento (não isolado) do zumbido com protocolo semanal (8 semanas), duração de 30 minutos cada sessão e acompanhamento por meio do THI e EVA. Tais recomendações devem ser exploradas em ensaios clínicos, investigando os efeitos a curto e longo prazo, em prol de evidências científicas e padronização de protocolos clínicos.

## INTRODUÇÃO

O zumbido é um sintoma definido como a percepção consciente de som na ausência de uma fonte geradora externa^(1)^. Sua prevalência varia conforme populações e estudos. Uma revisão sistemática e meta-análise indicou uma prevalência global de zumbido em adultos de aproximadamente 14,4%, com um aumento associado à idade^(2)^.

O zumbido pode estar associado a cerca de 300 condições clínicas^(3)^, incluindo doenças otológicas, traumas de cabeça e pescoço, alterações cardiovasculares, doenças metabólicas, neurológicas, psiquiátricas, fatores odontológicos, efeitos colaterais de medicamentos e abuso de drogas.

O impacto do zumbido é heterogêneo^(4)^, a maioria das pessoas não se incomoda, e aproximadamente um quarto dos pacientes procura atendimento médico^(1)^. Na presença de perturbação emocional, alteração cognitiva e/ou excitação autonômica, acarretando modificações comportamentais e incapacidade funcional, o quadro pode ser classificado de distúrbio do zumbido^(5)^.

Uma avaliação individualizada pode influenciar significativamente a adesão e êxito ao tratamento do zumbido^(6)^, especialmente quando realizada em contexto multidisciplinar (como otorrinolaringologia, fonoaudiologia, fisioterapia e psicologia) com visões complementares.

A identificação de fatores específicos do paciente permite intervenções direcionadas, que melhoram a qualidade de vida e, consequentemente, a adesão ao tratamento. A heterogeneidade clínica do zumbido requer a estratificação de subgrupos de pacientes que respondem de maneira mais previsível a tratamentos específicos^(7)^.

Em alguns casos, o aconselhamento sobre o zumbido é suficiente para restaurar a qualidade de vida dos pacientes, contudo se, em até dois meses, não houver melhora significativa, devem ser propostos tratamentos adicionais, seja por meio de uma única terapia ou combinando mais de uma delas^(8)^.

A prática de *mindfulness* (atenção plena) tem sido investigada como opção terapêutica para o tratamento do zumbido, sobretudo para redução do estresse e da angústia associados à condição. Define-se como o esforço de estar atento intencionalmente, sem julgamentos, no momento presente^(4)^.

Acredita-se que a angústia do zumbido pode ser gerada por pensamentos negativos e a tentativa de evitação. A *mindfulness* trabalha a habituação e aceitação, tendo se mostrado uma intervenção não farmacológica eficaz^(9)^.

No tratamento do zumbido, o principal desafio é, na maioria das vezes, aliviar a angústia associada ao sintoma. As abordagens baseadas em *mindfulness* podem oferecer uma nova maneira de enfrentá-lo, sendo bastante distintas dos tratamentos audiológicos convencionais^(10)^.

A atenção plena (mindfulness) foi introduzida na medicina moderna ocidental por Jon Kabat-Zinn, para tratar dor crônica, mas atualmente ela já se mostrou eficaz no tratamento de estresse, ansiedade e depressão^(4)^. Diferentes abordagens incluem *Mindfulness Based Stress Reduction* (MBSR), a *Mindfulness Based Cognitive Therapy* (MBCT) e a *Mindfulness Based Health Promotion* (MBHP).

A aplicação dessa técnica ao zumbido tem trazido benefícios, como redução da gravidade do sintoma, do sofrimento psicológico e da incapacidade^(11)^, como também diminuição da angústia relacionada ao zumbido^(9)^.

A prática da *mindfulness* pode ajudar a mitigar os efeitos negativos do controle mental irônico, no qual a tentativa de suprimir sons emocionais pode, paradoxalmente, aumentar a saliência e persistência do zumbido. Assim, ela ajuda os pacientes a desenvolverem uma consciência aberta e estável, possibilitando que eles "permitam" a presença do zumbido sem lutar contra ele, o que pode reduzir a intrusividade e o impacto emocional do zumbido^(12)^.

A aplicação dessa terapia oferece uma abordagem não invasiva, acessível e potencialmente eficaz para o manejo do zumbido crônico, com benefícios que incluem a redução do sofrimento e a melhoria do bem-estar geral dos pacientes^(4,13)^.

No entanto, os protocolos usados são muito variáveis, assim, faz-se necessário estabelecer um consenso que defina quais grupos de pacientes são melhores candidatos à *mindfulness* no tratamento do zumbido; a quantidade média de sessões necessária para remissão dos sintomas e a frequência recomendada; e como deve ser feito o *follow-up* desses pacientes.

Uma das maneiras de criação dos consensos é por meio do método *Delphi* ^(14,15)^. Trata-se de um procedimento para estruturar a comunicação entre um grupo de especialistas sobre assuntos complexos, por meio de interações realizadas por questionários. Estes são acompanhados de *feedbacks* via *e-mail,* mantendo-se o anonimato das respostas dos participantes, na busca de um resultado em comum, por meio de cálculos estatísticos^(14)^.

Esse método permite reunir um conjunto de opiniões de especialistas separados geograficamente, o que pode favorecer resultados consistentes sobre temáticas complexas^(16)^. O método *Delphi* é considerado importante nas pesquisas científicas das áreas da saúde, pois os consensos podem ser utilizados para determinar a direção das pesquisas científicas^(14)^.

Assim, o objetivo da presente pesquisa foi desenvolver um consenso com *experts* otorrinolaringologistas, fonoaudiólogos e fisioterapeutas acerca dos critérios de recomendação e uso da *mindfulness* aplicada ao tratamento do zumbido. Além de verificar o conhecimento destes quanto ao uso da *mindfulness* no tratamento do zumbido; identificar quais grupos de pacientes seriam os melhores candidatos à *mindfulness* no tratamento do zumbido, a quantidade média de sessões necessária para remissão dos sintomas, a frequência e como deve ser feito o seguimento desses pacientes.

## MÉTODO

Trata-se de um estudo do tipo observacional, descritivo, quantitativo e transversal. Baseou-se nas recomendações do *Strengthening the Reporting of Observational Studies in Epidemiology* (STROBE). Aprovado pelo Comitê de Ética em Pesquisa do Centro de Ciências da Saúde-CCS sob o parecer de número 6.666.318.

A pesquisa foi realizada de forma remota e online, por meio de uma *websurvey* hospedada na plataforma digital *Google Forms,* gerando maior comodidade, rapidez e redução de custos. O período de coleta de dados foi de junho a dezembro de 2024.

A amostra foi por conveniência, a primeira etapa contou inicialmente com 23 especialistas brasileiros (otorrinolaringologistas, fonoaudiólogos e fisioterapeutas) com reconhecido conhecimento acerca do tema em estudo. Para seleção da amostra, foram identificados profissionais que atendessem a pelo menos um dos critérios: (a) publicação de artigos na área de zumbido nos últimos dez anos, (b) participasse de projeto de pesquisa nesta área nos últimos cinco anos, (c) tivesse atuação clínica na área, (d) especialização relacionada ao zumbido, e/ou (e) conhecessem ou utilizassem a prática da *mindfulness* para tratamento do zumbido.

Na segunda etapa, enviou-se o questionário para 11 dos 23 especialistas (e obteve-se 10 respostas), pois adotou-se o critério de elegibilidade àqueles que, de fato, aplicavam a *mindfulness* nas suas práticas diárias. Entre os elegíveis, sete baseavam sua prática em uma abordagem específica de *mindfulness*, enquanto três não seguiam nenhum protocolo específico. Os demais apenas recomendam aos pacientes que façam e encaminham para profissionais especializados e isso poderia provocar um viés de confusão no nosso estudo.

A pesquisa utilizou o método *Delphi* para alcançar o consenso entre especialistas sobre o uso da *mindfulness* em pacientes com zumbido. Para tanto, foram seguidas algumas etapas para os procedimentos, descritas na Figura 1.

**Figura 1:** Etapas da pesquisa

Legenda: MCVCI = média dos coeficientes de validade de conteúdo dos itens do questionário; MPEI = média dos erros dos itens do questionário

A elaboração dos itens do primeiro questionário foi precedida de uma revisão da literatura. O instrumento de coleta de dados aplicado na primeira etapa incluiu questões subjetivas e objetivas que contemplavam os dados do perfil profissional dos participantes, bem como 14 questões sobre o uso da *mindfulness* no tratamento do zumbido.

Antes de responder às questões, estabeleceu-se contato com os participantes e disponibilizou-se o acesso ao texto explicativo sobre a pesquisa, a seguir, eles procederam com a leitura do TCLE e, caso estivessem de acordo, prosseguiam com as respostas. Após recebimento das respostas do questionário 1, foi realizada análise qualitativa, quantitativa e revisão destas.

Na segunda etapa, construiu-se o questionário 2 e forneceu-se os *feedbacks* via *e-mail* aos participantes sobre as perguntas da primeira etapa. O objetivo foi compreender como os profissionais utilizam a *mindfulness* para tratamento do zumbido nas suas práticas clínicas. Dos dez participantes da segunda etapa, todos tinham formação em *mindfulness*, porém sete baseavam sua prática em alguma abordagem específica de *mindfulness e* três não seguiam nenhum protocolo específico.

As 34 questões do questionário 2 foram todas objetivas, compostas por afirmativas surgidas nas respostas da primeira etapa. Estas afirmações foram inseridas em um formulário do *Google Forms* e solicitou-se aos especialistas que as classificassem utilizando a escala de *Likert* de cinco pontos, que oferece como opção de resposta nos extremos “discordo totalmente” (que corresponde a um ponto) a “concordo totalmente” (5 pontos) (Marques; Freitas, 2018).

Após responder a todas as questões, o especialista clicava em “Enviar” e o procedimento de coleta era finalizado. O tempo para concluir o formulário foi estimado em cerca de 10 minutos. O instrumento ficou disponível para respostas entre os dias 05 de novembro de 2024 a 06 de dezembro de 2024.

As respostas dos 10 especialistas para as 34 perguntas foram organizadas em uma planilha do *Microsoft Excel*, com o objetivo de realizar a análise de consenso. Foi realizada a análise de coeficiente de validade de conteúdo (CVC) para investigar o grau de concordância entre os juízes na avaliação de cada item do questionário, proposto por Hernández-Nieto (Hernández-Nieto, 2002). Com o cálculo do CVC, obteve-se as médias das notas dos especialistas na escala *Likert* (1 a 5 pontos para cada item).

Para análise, foi realizado cálculo da média das atribuições por item, ao total e de erro. Para o cálculo final, foi utilizado o CVC de cada item subtraído pelo constante da fórmula ou cálculo do erro (Figura 2). Os valores finais do CVC foram classificados como: (a) excelente (IVC ≥ 0,78), (b) bom (0,60 ≥ IVC ≤ 0,77); e (c) ruim (IVC ≥ 0,59) (I. *American Psychological Association*. II. *National Council on Measurement in Education*. III. *Joint Committee on Standards for Educational and Psychological Testing* (U.S.), 2014).

**Figura 2:** Fórmula utilizada para calcular o Coeficiente de Validade de Conteúdo (CVC)

Fonte: Honorato (2025)

Existem diversas definições para consenso (porcentagem de concordância, medida de tendência central, dispersão de respostas, entre outras), e há diferentes níveis para cada uma delas. Para esta pesquisa, adotou-se o critério de porcentagem de concordância, um nível acima de 70% que é amplamente considerado como rigoroso^(14)^.

## RESULTADOS

Participaram dessa pesquisa (questionário 1) 23 especialistas, cujas informações podem ser conferidas na Tabela 1.

**Tabela 1:** Caracterização profissional dos especialistas

**Variável**	**N**	**%**
**Profissão**		
Otorrinolaringologista	10	43,4
Fonoaudiólogo (a)	10	43,4
Fisioterapeuta	3	13
**Sexo**		
Feminino	20	86,9
Masculino	3	13
**Faixa etária**		
Entre 31 e 40 anos	10	43,4
Entre 41 e 50 anos	7	30,4
Entre 51 e 60 anos	6	26
**Escolaridade**		
Especialização	7	30,4
Mestrado	4	17,3
Doutorado	9	39,1
Pós-doutorado	3	13
	23	100

Fonte: Honorato (2025)

Na primeira etapa, todos os participantes conheciam a aplicação da *mindfulness* no tratamento do zumbido, porém apenas 34,78% tinham formação nessa área específica.

Quando questionados sobre se o entrevistado possuía publicação ou experiência no tratamento do zumbido, 86,9% responderam que sim. Enquanto três pessoas (13,1%) possuíam conhecimento na prática da técnica do *mindfulness*.

A maioria (82,6%) dos entrevistados recomendam a *mindfulness* na sua prática clínica para o tratamento do zumbido, porém apenas onze (47,8%), de fato, aplicam a técnica na sua prática diária.

Embora todos os especialistas afirmem que este é um recurso terapêutico indicado para o tratamento do zumbido, 56,5% não consideram que ele possa ser aplicado para todos os tipos. Quando perguntados sobre as três principais causas para zumbido que os especialistas mais fazem ou indicam o uso da *mindfulness*, as respostas mais frequentes foram: zumbido associado a causas emocionais ou a quadros depressivos ou ansiosos, zumbido somatossensorial, metabólico, associado à perda auditiva, entre outras.

Além disso, 82,6% dos entrevistados não utilizam a *mindfulness* como único tratamento (monoterapia) para esta indicação. As terapias complementares mais citadas foram: aconselhamento incluindo orientação alimentar, mudanças no estilo de vida e controle de comorbidades, reabilitação auditiva, terapia sonora, tratamento fisioterapêutico, uso de medicamentos, entre outras.

Dos onze especialistas que aplicam a *mindfulness* na sua prática clínica, foram obtidos os seguintes resultados (questionário 2) em relação ao protocolo que usam, a quantidade de sessões por semana, a duração das sessões, quantos exercícios faz em cada sessão, a quantidade mínima de sessões para atingir a remissão e como fazem o seguimento dos pacientes (Tabela 2).

**Tabela 2:** Respostas dos especialistas que aplicam *mindfulness* (questionário 1)

	**Protocolo**	**Sessões semanais**	**Duração sessão (minutos)**	**Exercícios por sessão**	**Número mínimo sessões para remissão**	**Seguimento**
1	MBCT	1	50	2	8	THI.
2	MBSR	1	60	2	8	Orientação e recomendação de como lidar com os desafios utilizando as ferramentas do *mindfulness.*
3	MBSR	7	10 a 15	1	Não sei	Não tenho protocolo de seguimento para *mindfulness.*
4	MBHP	2	60	3	8	Além de realizar alguns exercícios de *mindfulness* no consultório, o paciente segue com orientações de *mindfulness* indireto para realizar à domicílio onde eu tento trocar o estímulo à cada semana.
5	Não	7	60	Depende, cada sessão é única	Não sei	Especificamente, não sei.
6	Não	1	40	1	8	Consulta 1 vez por semana com orientações para casa. Dura 8 semanas.
7	MBCT	1	45	2 a 5	6 a 8	Ele fica com os áudios e todas orientações por escrito, sugiro que mantenha as mudanças diárias e pelo menos uma meditação diária.
8	Não	1	30	2	4	THI e EVA.
9	MBCT	1	60 a 90	3 a 4	Não sei	Semanal.
10	MBCT	1	40	1 a 2	8	Acompanhamento semanal com exercícios para casa e orientação para uso após última sessão.
11	MBCT	1	50	2	8	Estratégias para o uso da *mindfulness* no dia a dia.

Na segunda etapa do método *Delphi*, conseguiu-se obter respostas de dez dos onze especialistas que utilizavam *mindfulness* na prática. Considerando-se que o objetivo desta pesquisa é desenvolver um consenso acerca do uso da *mindfulness* no tratamento do zumbido, foram considerados consensuais apenas os itens que apresentarem um CVC acima de 0,70^(14)^. A partir dos resultados encontrados, pode-se observar que houve consenso em 25 dos 34 itens (CVC ≥ 0,70), que podem ser verificados na Tabela 3.

**Tabela 3:** Valores do Coeficiente de Validade de Conteúdo (CVC) para cada item do questionário

ITEM	CVC item
Eu acredito que a *mindfulness* é um recurso terapêutico indicado para o zumbido	0,979
Eu acredito que *mindfulness* possa ser aplicada a todos os tipos de zumbido	0,739
Eu NÃO acredito que *mindfulness* possa ser aplicada a todos os tipos de zumbido	0,519
Eu acredito que *mindfulness* pode ser aplicada para zumbido por causas emocionais ou psicogênicas ou relacionados à transtornos do humor	0,979
Eu acredito que mindfulness pode ser aplicada para zumbido por zumbido somatossensorial	0,859
Eu acredito que mindfulness pode ser aplicada para zumbido por disfunção de ATM (articulação temporo-mandibular)	0,859
Eu acredito que mindfulness pode ser aplicada para zumbido por perda auditiva	0,899
Eu acredito que mindfulness pode ser aplicada para zumbido que provoque impacto ou transtornos do sono e nas atividades diárias	0,979
Eu acredito que mindfulness pode ser aplicada para zumbido por síndrome do tensor do tímpano	0,819
Eu acredito que mindfulness pode ser aplicada para zumbido que tenha duração maior que 6 meses, independente da causa	0,859
Eu acredito que mindfulness pode ser aplicada para zumbido pulsátil	0,739
Eu acredito que mindfulness NÃO pode ser usado como única terapia no zumbido (monoterapia)	0,819
Eu acredito que mindfulness pode ser usado como única terapia no zumbido (monoterapia)	0,459
No tratamento mindfulness pode ser associada a aconselhamento ou psicoeducação sobre zumbido	0,979
No tratamento mindfulness pode ser associada a terapia cognitivo comportamental	0,979
No tratamento mindfulness pode ser associada a psicoterapia	0,979
No tratamento mindfulness pode ser associada a fotobiomodulação	0,939
No tratamento Mindfulness pode ser associada a tDCS (*Transcranial Direct Current Stimulation*)	0,939
No tratamento Mindfulness pode ser associada a TRT (Terapia de Retreinamento do Zumbido)	0,939
No tratamento mindfulness pode ser associada a treinamento auditivo	0,939
No tratamento mindfulness pode ser associada a manobras de fisioterapia (terapia manual, estimulação vagal)	0,959
No tratamento mindfulness pode ser associada a neuromodulação	0,959
No tratamento mindfulness pode ser associada a terapia medicamentosa	0,959
Aplico mindfulness uma vez por semana com profissional e oriento prática diária domiciliar	0,899
Aplico mindfulness duas vezes por semana com profissional e oriento prática diária domiciliar	0,359
As sessões de mindfulness duram em torno de 1h	0,579
As sessões de mindfulness duram em torno de 30 minutos	0,739
Realizo um exercício em cada sessão	0,739
Realizo dois exercícios em cada sessão	0,679
Realizo três ou mais exercícios em cada sessão	0,519
A quantidade mínima de sessões para remissão são 4 sessões	0,339
A quantidade mínima de sessões para remissão são 6 sessões	0,479
A quantidade mínima de sessões para remissão são 8 sessões	0,659
O seguimento da terapia após 8 semanas pode ser feito através de escalas como THI (*Tinnitus Handicap Inventory*) e EVA (Escala Visual Analógica)	0,979

Fonte: Honorato (2025)

## DISCUSSÃO

Métodos de consenso auxiliam a tomada de decisão clínica, definindo critérios em cenários com evidências externas limitadas ou incerteza sobre efetividade. O tratamento do zumbido é um desafio clínico, pois, além de existirem múltiplas possibilidades de etiologias e intervenções, deve-se considerar o impacto na qualidade de vida do paciente^(17)^.

A *mindfulness* tem se mostrado um tratamento eficaz para o zumbido, mas há variabilidade nos protocolos, quanto à quantidade de sessões e às estratégias de seguimento desses pacientes^(8,11,18)^. Este trabalho objetiva desenvolver um consenso para refinar as recomendações acerca do uso dessa estratégia no tratamento do zumbido.

### Itens consensuais entre os especialistas

#### 1) Melhores candidatos à mindfulness no tratamento do zumbido

Houve consenso entre os especialistas que a *mindfulness* é um recurso terapêutico indicado para o zumbido (CVC 0,979). Uma revisão sistemática avaliou a eficácia das intervenções baseadas em *mindfulness* no alívio da angústia causada pelo zumbido e encontrou redução significativa da angústia relacionada ao zumbido^(4)^ e a MBCT foi associada a maior aceitação do sintoma^(10)^.

Também foi consenso que a *mindfulness* pode ser aplicada a todos os tipos de zumbido (CVC 0,739), sem distinção da característica sonora, incômodo e/ou duração. Em ordem decrescente de níveis de concordância tivemos: zumbido por causas emocionais ou psicogênicas ou relacionados à transtornos do humor e zumbido que provoque impacto ou transtornos do sono e nas atividades diárias (CVC 0,979), zumbido por perda auditiva (CVC 0,899). E também zumbido somatossensorial, por disfunção de ATM (articulação temporo-mandibular) e zumbido que tenha duração maior que 6 meses, independente da causa (CVC 0,859). Além de zumbido por síndrome do tensor do tímpano (CVC 0,819) e zumbido pulsátil (CVC 0,739).

Embora os estudos que tratam do uso da *mindfulness* no tratamento do zumbido explorem pouco a causa específica do zumbido, eles indicam que essa terapia pode ser aplicada a diferentes tipos de zumbido, desde que o foco seja o alívio do sofrimento psicológico e a melhoria da qualidade de vida dos pacientes. No entanto, as abordagens terapêuticas direcionadas às hipóteses etiológicas estão mais próximas do sucesso no tratamento^(19)^.

Por outro lado, a eficácia dessas intervenções não parece ser limitada por características específicas do zumbido, como a sua origem ou tipo, mas sim pela capacidade do paciente de se engajar na prática de *mindfulness* e desenvolver uma nova relação com o zumbido^(11,12)^.

#### 2) Terapias associadas

Houve concordância que a *mindfulness* não pode ser usada como única terapia no zumbido (CVC 0,819). A combinação de terapias para o tratamento do zumbido pode oferecer benefícios superiores em comparação com terapias isoladas, especialmente quando individualizadas com base nas características do paciente. Tal como demonstrou uma meta-análise^(20)^ que indicou que a terapia combinada, como a estimulação sonora associada à consulta educacional ou à terapia medicamentosa, apresentou resultados significativamente melhores na redução do zumbido em comparação com tratamentos individuais.

Além disso, terapias combinadas parecem mais adequadas no tratamento do zumbido, uma vez que as evidências, na maioria das vezes, não são suficientes para apoiar um único método de tratamento. E também abordagens diferentes somam e complementam os mecanismos pelos quais promovem uma melhora do zumbido^(21)^.

No que concerne às terapias que podem ser associadas à *mindfulness,* obtiveram maior índice de concordância (CVC 0,979): aconselhamento ou psicoeducação sobre zumbido, terapia cognitivo comportamental e psicoterapia. Seguidas por manobras de fisioterapia (terapia manual, estimulação vagal), neuromodulação e terapia medicamentosa (CVC 0,959). E, também, fotobiomodulação, *Transcranial Direct Current Stimulation* (tDCS), Terapia de Retreinamento do Zumbido (TRT) e treinamento auditivo (CVC 0,939).

A psicoeducação em zumbido consiste em fornecer informações aos pacientes sobre a natureza do zumbido, as causas possíveis e as melhores formas de lidar com ele. Em alguns casos, essa terapia é suficiente para restaurar uma qualidade de vida normal em pessoas com zumbido, não sendo necessárias outras intervenções^(8)^. O consenso alemão de zumbido crônico sugere que a psicoeducação do paciente deve ser o ponto de partida de qualquer intervenção terapêutica com base em uma avaliação diagnóstica exaustiva^(22)^.

As abordagens terapêuticas que incluem aconselhamento, como a Terapia Cognitivo-Comportamental (TCC), a Terapia de Aceitação e Compromisso (ACT), a *mindfulness* e a Terapia de Retreinamento do Zumbido (TRT), têm demonstrado maior eficácia no manejo do zumbido, sendo, portanto, recomendadas como prioridade^(22,23)^.

Além disso, terapias sonoras, como o uso de mascaradores ou aparelhos auditivos, são frequentemente recomendadas. Porém, há variações nas orientações sobre o uso de medicamentos e terapias alternativas, refletindo a falta de evidências conclusivas em algumas áreas^(22,23)^.

Nos casos em que o zumbido está associado a disfunções da articulação temporomandibular ou problemas cervicais, terapias manuais e fisioterapia mostram-se estratégias promissoras, sendo indicadas dentro de uma abordagem multidisciplinar e personalizada^(24)^.

#### 3) Frequência, duração e quantitativo de exercícios

Em relação à frequência de aplicação dessa técnica, foi obtido consenso na indicação de uma vez por semana com prática diária domiciliar. Todos os protocolos citados, na primeira etapa pelos especialistas, utilizam a frequência de sessões semanais com o profissional e orientação para realizar exercícios diariamente domiciliar, com duração total de oito semanas^(25)^.

Nos ensaios clínicos randomizados que investigaram a eficácia da MBCT para o tratamento do zumbido, houve variações na duração do tratamento. No estudo de McKenna *et al.*, a MBCT foi administrada em 8 sessões, semanalmente, cada uma com 120 minutos de duração^(10)^. Já Gans *et al.* também manteve a estrutura de 8 sessões, mas com durações de 150 minutos cada^(26)^.

Por outro lado, os estudos de Philippot *et al.* e Arif *et al.* utilizaram formatos diferentes, com respectivamente seis e cinco sessões de *mindfulness,* semanalmente^(8,18)^. Essas abordagens em períodos mais condensados também mostraram benefícios na redução das dificuldades psicológicas associadas ao zumbido.

Em relação à duração das sessões, o consenso obtido foi sessões de aproximadamente 30 minutos. Nos protocolos MBSR e o MBCT, a duração das sessões sugeridas é de 2h a 2h30. Já no MBHP, a duração é de 90 minutos e incluem práticas diárias de meditação de 45 minutos como parte fundamental do processo^(25)^.

Embora, no consenso atingido tenha sido de aproximadamente 30 minutos em relação à duração das sessões, na primeira etapa do estudo, a maioria dos pesquisadores responderam que suas sessões costumam durar uma média de 49 minutos (como pode ser visto na Tabela 2). Assim, entende-se que a terapia deve ter duração mínima de 30 minutos.

No que concerne à quantidade de exercícios por sessão, o item 1 exercício por sessão obteve índice de 0,739. Os ECRs que usam a *mindfulness* no tratamento do zumbido que melhor especificam os exercícios que realizam em cada sessão foram os estudos de Arif *et al.* (2017) e Philippot *et al.* (2011).

O primeiro^(18)^ descreve que as aulas começavam com uma prática de meditação de 40 minutos, seguida de uma revisão do trabalho de casa da semana anterior, apresentação e discussão do tema dessa sessão, e terminava com um exercício experimental mais curto e uma revisão do trabalho de casa para a semana seguinte.

Já o segundo^(8)^, detalhou em uma tabela tudo o que faz em cada sessão, que começava com um exercício de 30 minutos e continuava com a revisão dos trabalhos de casa da semana anterior, antes de apresentar e discutir o tema específico da sessão. Por fim, todas as sessões terminavam com um exercício experimental.

#### 4) Seguimento (follow-up) dos pacientes

Também se obteve consenso que o seguimento da terapia pode ser feito por meio de escalas como THI (*Tinnitus Handicap Inventory*) e EVA (Escala Visual Analógica).

Os métodos de avaliação do zumbido são ferramentas essenciais para caracterizá-lo e documentar a eficácia dos tratamentos. Atualmente, os mais comuns são: THI e EVA*.* O THI tem como vantagem ser uma avaliação mais abrangente, por outro lado, a EVA é mais simples e mais fácil de assimilar^(27)^.

O THI é um questionário composto por 25 itens que avaliam o impacto do zumbido em três dimensões: funcional, emocional e catastrófica. Ele foi desenvolvido para medir a severidade do zumbido e o grau de incômodo que ele causa, fornecendo um escore total que reflete a gravidade global do zumbido. Estudos indicam que o THI é uma escala confiável^(28)^.

A EVA é uma ferramenta simples e rápida que mede o sofrimento subjetivo causado pelo zumbido. Ela avalia aspectos como a intensidade do som, a consciência do zumbido, o incômodo e o impacto na vida do paciente e é eficaz para capturar mudanças rápidas na percepção do zumbido ao longo do tempo^(29)^. Ela também é considerada útil para uma avaliação inicial da gravidade do zumbido e tem mostrado boa correlação com outras medidas de severidade do zumbido, como o THI^(30)^.

Gans *et al*. utilizaram, em seu estudo, o THI como medida de resultado primário e o consideraram fácil de administrar, interpretar e psicometricamente robusta. E incluíram a EVA, além de outros questionários para as medidas de resultados secundários.

Mckenna *et al*. e Arif *et al*. utilizaram a EVA dentre os questionários utilizados para medir a gravidade percebida do zumbido e as melhorias pós-tratamento, tanto nos grupos tratados com *mindfulness* quanto nos grupos controle. Já Philippot *et al.* utilizaram um questionário não publicado e não validado até o momento da sua publicação.

Roland *et al*. realizaram um ensaio clínico não controlado envolvendo 13 pacientes com zumbido crônico incômodo, que foram submetidos à MBSR. A medida de desfecho primário utilizada foi a diferença nos sintomas de zumbido relatados pelo paciente usando o THI e *o Tinnitus Functional Index* (TFI) entre as avaliações pré-intervenção, pós-MBSR e 4 semanas pós-MBSR^(13)^.

Em relação à duração, esse estudo não fez essa avaliação entre os especialistas. Philippot *et al.* e Arif *et al.* , acompanharam os pacientes por 3 meses. Já Mckenna *et al.* o fez 1 mês após e após 6 meses, demonstrando que os efeitos da *mindfulness* persistiram por longos períodos.

Um estudo avaliou 80 pacientes com remissão total do zumbido, fez o seguimento semestral dos pacientes até completar 18 meses. Durante este período, 92,1% dos participantes permaneceram assintomáticos, enquanto apenas 7,9% relataram recorrência do sintoma^(21)^. Esses achados incentivam uma postura mais otimista na condução dos pacientes que sofrem com zumbido, sugerindo que a personalização das estratégias terapêuticas pode levar ao êxito em muitos casos.

As diretrizes mais recentes publicadas para manejo do zumbido em diversos continentes não abordam recomendações sobre como deve ser feito o seguimento desses pacientes^(17,22,23)^. Dessa forma, conforme o consenso, pensamos ser importante acompanhar os pacientes utilizando, pelo menos, o THI e a EVA.

### Itens não consensuais entre os especialistas

Não há um consenso entre os especialistas sobre a possibilidade de remissão do zumbido, bem como da quantidade mínima de sessões necessárias. É importante destacar que a definição de remissão na literatura é controversa. Gouveia considerou que a remissão ocorre quando o paciente que tinha zumbido por mais de 3 meses diariamente deixou de percebê-lo por, pelo menos, 6 meses^(31)^. No entanto, tal definição pode não ser aplicável a todos os contextos terapêuticos, especialmente àqueles voltados para abordagens de manejo do sintoma, como é o caso da *mindfulness*.

Além disso, a resposta terapêutica varia significativamente entre os pacientes. E também uma parcela dos pacientes alcança uma habituação ao sintoma em vez de sua eliminação completa. Outro fator relevante é que a maioria dos especialistas não aplica *mindfulness* como única terapia, de forma que os casos de sucesso podem ser atribuídos a outras técnicas ou a combinação entre elas.

O estudo apresenta limitações quanto às respostas serem limitadas à opinião de alguns especialistas, além de terem sido questionados por meio de perguntas fechadas, limitando a discussão sobre o assunto. Tal decisão foi realizada devido à necessidade de expandir a coleta de dados sem limitar as barreiras geográficas e facilitar a participação.

Outra limitação refere-se à necessidade da realização de uma terceira etapa, com os tópicos que não alcançaram concordância, para tentar ampliar o nível de consenso por meio de *feedback* e as avaliações de cada indivíduo. Essa interação não foi possível devido à fadiga da amostra. Os itens que dizem o oposto, em relação aos tópicos já discutidos, não obtiveram consenso, tal como reforçou-se com a literatura e o consenso o que deve ser utilizado.

## CONCLUSÃO

A *mindfulness* é um recurso terapêutico indicado para o zumbido, porém não de forma isolada. Em relação à frequência de aplicação dessa técnica, foi obtido consenso à indicação de uma vez por semana com terapeuta, com duração de, pelo menos, 30 minutos, somada a prática diária domiciliar. No que concerne à quantidade de exercícios por sessão, o item de um exercício por sessão obteve consenso. Também houve concordância de que o seguimento da terapia, após oito semanas, pode ser avaliado por meio de questionários e/ou escalas, como THI e EVA. O único tópico sobre o qual não houve consenso foi a quantidade mínima de sessões para possibilitar uma possível remissão do zumbido.
